# Effects of Difluoro(oxalato)borate-Based Ionic Liquid as Electrolyte Additive for Li-Ion Batteries

**DOI:** 10.3390/ma16041411

**Published:** 2023-02-08

**Authors:** Graziano Di Donato, Giovanna Maresca, Matteo Palluzzi, Akiko Tsurumaki, Maria Assunta Navarra

**Affiliations:** Department of Chemistry, Sapienza University of Rome, Piazzale Aldo Moro 5, 00185 Rome, Italy

**Keywords:** lithium batteries, ionic liquids, batteries

## Abstract

In this work, the use of *N*-methyl-*N*-propylpiperidinium difluoro(oxalato)borate Pip_13_DFOB ionic liquid (IL), originally synthesized in our laboratory, as an additive for liquid electrolytes in lithium-ion batteries (LIBs), is proposed. The synthesized IL exhibits glass and melting transitions at −70.9 °C and 17.1 °C, respectively, and a thermal decomposition temperature over 230 °C. A mixture based on 1.0 M LiPF_6_ in 1:1 *v*/*v* ethylene carbonate (EC): dimethyl carbonate (DMC) electrolyte solution (so called LP30) and the IL was prepared and tested in lithium metal cells versus two different commercially available carbonaceous electrodes, i.e., graphite (KS6) and graphene (GnP), and versus a high voltage LiNi_0.5_Mn_1.5_O_4_ (LNMO) cathode. A noticeable improvement was observed for Li|LNMO cells with an IL-added electrolyte, which exhibited a high specific capacity above 120 mAh g^−1^ with a Coulombic efficiency above 93% throughout 200 cycles, while the efficiency fell below 80% after 80 cycles with the absence of IL. The results confirm that the IL is promising additive for the electrolyte, especially for a longer cycle life of high-voltage cells.

## 1. Introduction

Lithium-ion battery (LIB) technology is currently dominating the market of energy storage systems, and there is still increasing demand for higher performing batteries. The use of high voltage cathodes, such as LiNi_0.5_Mn_1.5_O_4_ (LNMO), is known to be essential for boost in energy density delivered by LIBs [1,2]. In the meantime, the safety of electrolytes is recognized as a critical issue for ensuring LIB reliability. Typically, electrolytes for LIBs consist of one (or more) lithium salt(s) dissolved in organic aprotic solvents, such as carbonate and ether, in order to guarantee high ionic conductivities, high Li^+^ transference number, and good electrochemical stability. However, the presence of these solvents in the electrolytes potentially brings safety concerns related to their flammability [3,4]. Therefore, a crucial strategy in development of new electrolytes is the partial or complete replacement of the flammable solvents with materials having lower flammability and minor vapor pressure [5,6].

In this regard, ionic liquids (ILs) emerged as very promising electrolyte components for assuring battery safety [7,8]. Generally, room-temperature ILs (RTIL) consist of an organic cation and charge-delocalized anion. Among this type of ILs, strong Coulomb interaction between the cations and anions is substantially reduced by the charge-delocalized characteristics of anions, which leads to unique RTIL properties: (i) low melting point below 100 °C and (ii) high ionic conductivity, but still (iii) non-volatile and non-flammable ensuring thermal stability. In addition, ILs are known as a designable solvent because of the wide variety in their ionic structures [9,10,11]. Imide-based anions, such as bis(trifluoromethanesulfonyl)imide (TFSI) and bis(fluorosulfonyl)imide (FSI), are the most frequently used anions in battery applications because of the high ionic conductivity of resulting ILs [12,13]. Recently, borate-based anions, such as bis(oxalato)borate (BOB) and difluoro(oxalato)borate (DFOB), are also garnering attention owing to their ability to form a solid electrolyte interphase (SEI) on cathodes, so-called CEI [14,15,16]. Between these two borate-based anions, ILs with [BOB] anions generally yield as a solid due to a symmetric anion structure. In contrast to this, ILs with [DFOB] anions with charge-delocalized structure induced by fluorine atoms tend to be liquid, similar to the case of [TFSI] and [FSI] anions [17].

Recently, the use of *N*-methyl-*N*-propylpyrrolidinium difluoro(oxalato)borate, Pyr_13_DFOB, as an electrolyte component in high-voltage supercapacitor is reported [18]. In the same article, synthesis of ILs is carried out by mixing *N*-methylpyrrole, lithium difluoro(oxalato)borate, and 1-bromopropane in acetonitrile. In contrast to this, in this study, *N*-methyl-*N*-propylpiperidinium difluoro(oxalato)borate, Pip_13_DFOB, was prepared through a two-step synthesis procedure, specifically a quaternization of amine and an anion-exchange reaction with halide salts. The IL was added to commercially available electrolyte solution, 1.0 M LiPF_6_ in 1:1 *v*/*v* ethylene carbonate (EC):dimethyl carbonate (DMC) solution (LP30). The effect of the presence of IL on the battery performance was evaluated, especially in the lithium metal cells, having either a high-voltage LNMO cathode or carbonaceous electrodes, in order to evaluate the formation of CEI and SEI, respectively.

## 2. Materials and Methods

### 2.1. Synthesis of Pip_13_DFOB IL

*N*-methylpiperidine, 1-bromopropane, and lithium difluoro(oxalato)borate (LiDFOB) were purchased from Merck KGaA (Darmstadt, Germany) and used without further purifications. Equimolar amounts of *N*-methylpiperidine and 1-bromopropane were mixed in acetonitrile, twice their mass combined, under an N_2_ atmosphere. The mixture was stirred under reflux at 80 °C for 24 h. After the evaporation of acetonitrile, an orange solid was obtained, which was then mixed with activated carbon in methanol overnight. A yellow solid was obtained after the evaporation of a filtered sample. Recrystallization of the sample was performed in the ethyl acetate and acetonitrile 1:2 *v*/*v* solution to obtain a white solid. The yield of Pip_13_Br was 78%.

**Figure 1:**
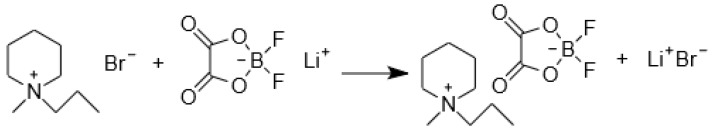
Scheme of the metathesis reaction.

Pip_13_DFOB was obtained through a salt metathesis reaction between Pip_13_Br and LiDFOB (Figure 1). Firstly, Pip_13_Br and LiDFOB in a 1:1.1 molar ratio were mixed in de-ionized water (DIW). To this solution, 3 times the volume of dichloromethane (DCM) was added to create two immiscible phases. The DCM phase containing a crude IL was collected while the aqueous phase underwent two additional extraction steps using the same amount of DCM to maximize the recovery of the IL. The IL–DCM solution was then washed three times with DIW, and the IL was obtained after the removal of DCM by using a rotary evaporator. The absence of bromide anion impurities was confirmed by checking the absence of silver halide precipitate when a drop of the ILs was mixed with AgNO_3_/HNO_3_ solution. Finally, IL diluted in DCM was passed through a column filled with aluminum oxide (neutral, Brockamann from Merck KGaA, Darmstadt, Germany) for a further purification, and the purified IL was obtained by drying under vacuum at 70 °C for 12 h (Glass Oven B-585, Büchi, Flawil, Switzerland). The final yield was about 68%.

The chemical structure of the obtained compound was confirmed by ^1^H NMR (Bruker 400 MHz) (Figure S1): ^1^H NMR (400 MHz, DMSO-d_6_) δ 3.27–3.30 (m, 4H), 3.24 (q, *J* = 4.2 Hz, 2H), 2.98 (s, 3H), 1.78 (t, *J* = 5.1 Hz, 4H), 1.63–1.73 (m, 2H), 1.47–1.61 (m, 2H), 0.91 (t, *J* = 7.3 Hz, 3H)].

### 2.2. Preparation of Electrolyte Mixture

A commercially available Li-ion-conducting electrolyte, composed of 1.0 M LiPF_6_ in 1:1 *v*/*v* EC:DMC electrolyte solution, so called LP30, was purchased from Solvionic (Toulouse, France). Using this electrolyte, 0.1M IL solution was prepared in an Ar-filled glovebox (UNIlab workstation, MBraun, München, Germany). The solution was then stirred overnight.

### 2.3. Thermal Analyses

Thermogravimetric analysis (Mettler-Toledo TGA2, Mettler-Toledo, Columbus, OH, USA) was performed under nitrogen flow from room temperature to 600 °C with a heating rate of 10 °C min^−1^. Differential scanning calorimetry (Mettler-Toledo DSC 821, Mettler-Toledo, Columbus, OH, USA) was performed with the following procedure: the sample was cooled from room temperature until −120 °C with a −10 °C min^−1^ rate, and then the heating scan was recorded from −120 °C until 140 °C with a rate of 5 °C min^−1^.

### 2.4. Electrochemical Investigations

#### 2.4.1. Preparation of Electrodes

Active materials such as LNMO (NEI Corporation, Somerset, NJ, USA), KS6-graphite (TIMREX KS-6, Timcal, Bodio, Switzerland), and GnP-graphene nanoplates (xGnP Graphene Nanoplates, from Merck KGaA, Darmstadt, Germany) were purchased and used as received. Electrode slurries based on *N*-methylpyrrolidone (NMP) containing a mixture of the active material, conductive carbon (Super-P, Timcal, Bodio, Switzerland), and polyvinylidene fluoride (PVDF 6020, Solvay, Brussels, Belgium) as binder in 80:10:10 (wt.%) ratio were prepared and coated on Al- or Cu-based current collectors. After removal of NMP, the electrodes were cut into a disk with 1 cm diameter and dried at 120 °C under vacuum for overnight. A 200 µm thick lithium was purchased from Chemetall foote corp.

#### 2.4.2. Impedance Spectroscopy

Ionic conductivity of pure IL, LP30, and their mixture was evaluated in the temperature range of 25–70 °C by means of electrochemical impedance spectroscopy (VSP Bio-Logic, Seyssinet-Pariset, France). The cell was prepared by immersing a pair of Pt electrodes (standard cells for conductivity, AMEL S.r.l., Milan, Italy) in the sample solutions. The frequency range was set to 1.0 MHz–1.0 Hz with a signal amplitude of 10 mV. The interval of measurements was set to 6 h in order to stabilize the temperature. All procedures were performed in an Ar-filled glove box.

**Figure 2:**
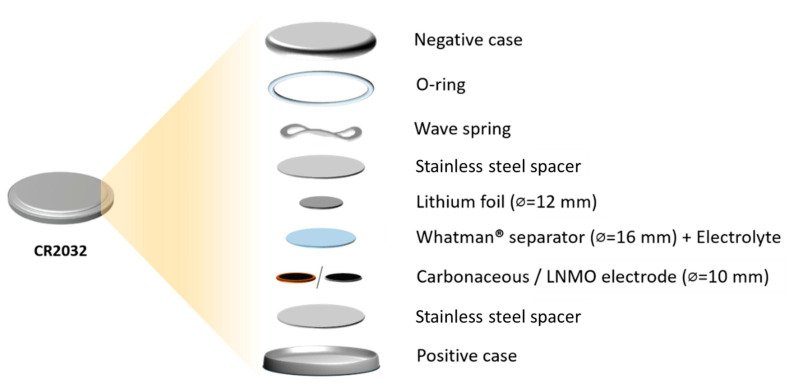
Coin cell configuration for galvanostatic cycling.

For the analysis of long-term stability of the interphase between the electrode and electrolytes, KS6|KS6, Li|Li, and LNMO|LNMO symmetric cells with LP30 or LP30 containing 0.1 M Pip_13_DFOB solution were assembled. For this analysis, coin cell CR2032 configuration, reported in Figure 2, was used. The impedance spectra were recorded in the frequency range of 100 kHz–100 Hz with 10 mV of a signal amplitude.

#### 2.4.3. Galvanostatic Cycling

The electrochemical performance in terms of capacity was analyzed by galvanostatic cycling. Lithium metal cells with the CR2032 configuration were assembled in an Ar-filled glovebox and tested at room temperature using a Maccor Series 4000 Battery Test System (Maccor, Tulsa, OK, USA). One piece of Whatman GF/F soaked in 120 µL of electrolyte was used as separator between the electrodes. The charge and discharge potentials were limited between 5.0–3.5 V (vs. Li^+^/Li) for the cell with LNMO, 2.5–0.05 V for KS6, and 3.0–0.05 V for GnP. The former cell was cycled at 1C (=0.41 mA), while latter two were cycled at 0.1C (=0.03 mA).

## 3. Results and Discussion

### 3.1. Thermal Properties

**Figure 3:**
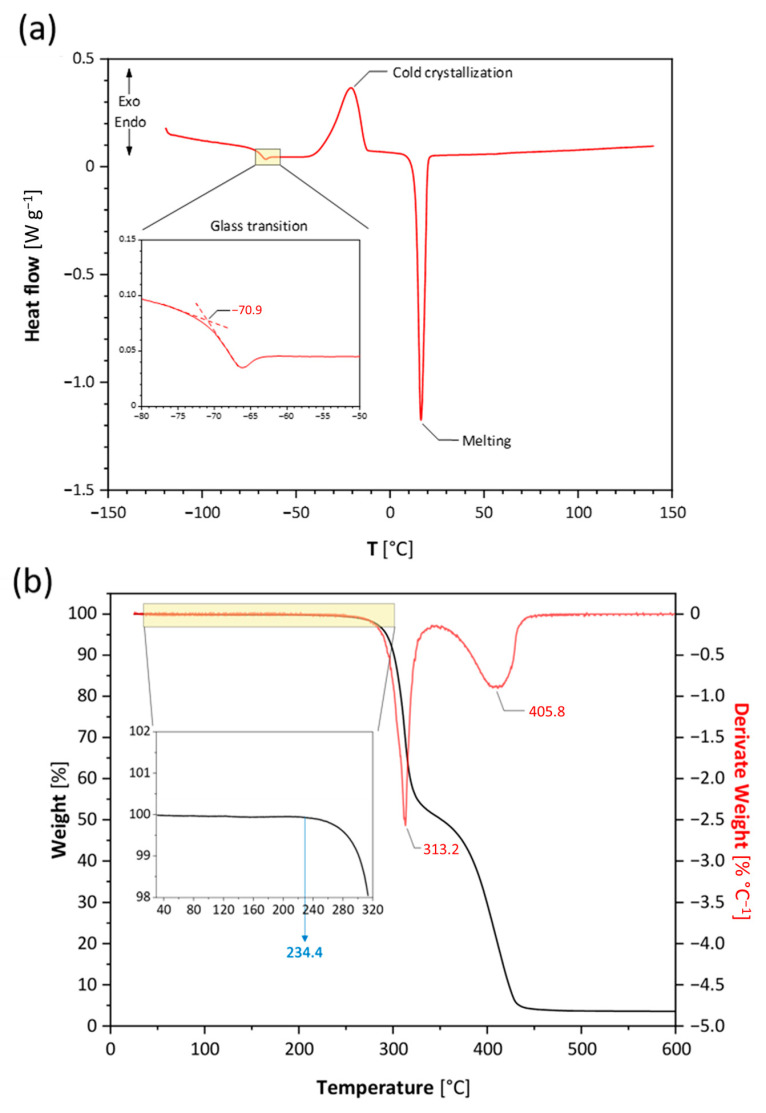
DSC heating scan (**a**), TGA curve (**b**, black) and its derivate (**b**, red) of Pip_13_DFOB.

In order to investigate characteristic phase transitions of the IL, a DSC analysis was carried out. In the thermogram of heating scan (Figure 3a), three different peaks are observed, of which two exothermic processes relate to the glass transition (T_g_) and the melting point (T_m_), while an endothermic one is assigned to the cold crystallization. The T_g_ value is identified by the extrapolated onset temperature given by the interception of the tangent lines of the peak (insert of Figure 3a) and is found to be −70.9 °C. The obtained value is comparable to that of other [DFOB]-based ILs [14]. The T_m_ of 17.1 °C confirms that Pip_13_DFOB is a RTIL. By calculating the area of the endothermic peak, enthalpy of fusion (ΔH_m_) is found to be about 52.6 J g^−1^.

The thermal stability of the IL was investigated through TGA analysis (Figure 3b). The decomposition temperature (T_d_) of 234.4 °C is determined based on the onset of the weight loss curve. The sample has no loss of weight below this temperature, confirming the absence of other volatile impurities such as the solvents used during the synthesis and purification of the IL. The first derivate curve shows two separate processes at 313.2 °C and 405.8 °C. As already pointed out in the literature [14,19], these peaks can be assigned to the decomposition of oxalate borate and fluoroborate structures, respectively. Compared to the same literature, reporting an IL with [DFOB] combined with an ether-functionalized cation, the decomposition temperature of Pip_13_DFOB is slightly higher [14]. This suggests also the cation structure has an effect on the decomposition process. In any case, the first decomposition temperature is above 234 °C, confirming the outstanding thermal stability of IL in the temperature range wide enough for battery cycling.

### 3.2. Ionic Conductivity

**Figure 4:**
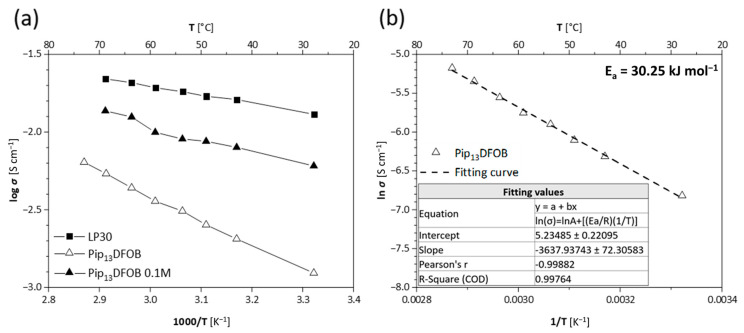
Ionic conductivity trend vs. temperature for Pip_13_DFOB, LP30, and 0.1 M Pip_13_DFOB solution (**a**), and a fitting result of conductivity of Pip_13_DFOB according to Arrhenius equation (**b**).

Figure 4a summarizes the temperature-dependence of ionic conductivity (σ) for the pure IL, LP30, and their mixture. At room temperature, the IL shows σ of about 1.24 × 10^−3^ S cm^−1^, being one magnitude lower than the LP30 solution but typical for RTILs [14,20,21,22]. At high temperatures, the conductivity of IL almost achieves 10^−2^ S cm^−1^, and the difference in the conductivity between Pip_13_DFOB and LP30 decreases. In the temperature range investigated, the σ of Pip_13_DFOB is properly fitted by using the Arrhenius equation (Figure 4b):

$$\ln\sigma =\ln A-\frac{E_{a}}{R}\left(\frac{1}{T}\right)$$

where A is a pre-exponential factor, E_a_ is the activation energy, R is the gas constant (8.314 J/mol·K), and T is the absolute temperature. The E_a_ calculated for the pure IL is 30.25 kJ mol^−1^. By mixing Pip_13_DFOB and LP30, the room temperature σ almost reaches 10^−2^ S cm^−1^, fulfilling the requirement as battery electrolytes.

### 3.3. Long-Term Stability at the Electrode Surface

**Figure 5:**
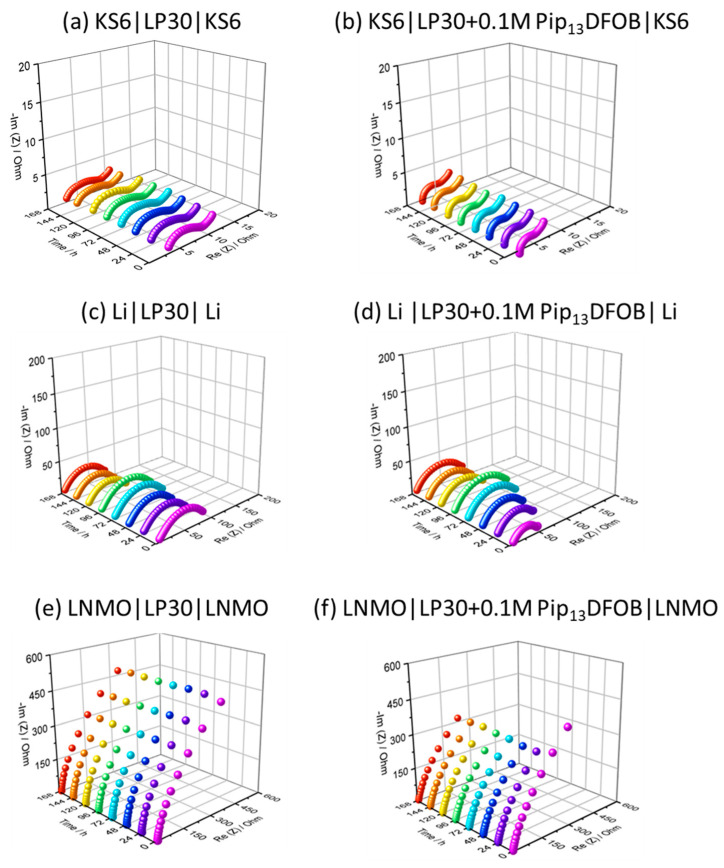
Nyquist plots of KS6|KS6 (**a**,**b**), Li|Li (**c**,**d**), and LNMO|LNMO (**e**,**f**) symmetric cells with LP30 (**a**,**c**,**e**) and 0.1M Pip_13_DFOB solution (**b,d**,**f**).

Figure 5 shows the evolution of interfacial resistances between the electrolyte and electrodes. For this evaluation, KS6|KS6, Li|Li, and LNMO|LNMO symmetric cells with either LP30 or LP30 containing 0.1M Pip_13_DFOB solution were assembled, and their impedance spectroscopy were recorded daily for 7 days. The high-frequency region (100 kHz–100 Hz), corresponding to bulk and interfacial resistances, was especially investigated. The resistance values obtained by fitting are reported in the Supplementary Materials (Tables S1–S3). For Li|Li cells, since only a suppressed semicircle is observed in each spectrum, the equivalent circuit, R_b_ + Q_int_/R_int_, in which R and Q are resistance and constant phase element, respectively, is used for fitting. In contrast, for KS6|KS6 and LNMO|LNMO cells, the equivalent circuits such as R_b_ + Q_int_/R_int_ + M and R_b_ + Q_dff_/W_dff_ were used to account for diffusion in the electrodes. The bulk resistances (R_b_) are found to be around 5 Ω except for a LNMO|LNMO cell with LP30. Even though the ionic conductivity of LP30 and its mixture with the IL is different (see Figure 4), the R_b_ are similar in KS6|KS6 as well as Li|Li cells. On the contrary to the conductivity values, the R_b_ is found to be smaller for the IL-added electrolyte in the case of the LNMO|LNMO symmetric cell.

When the KS6 is used as the electrode, small semicircles are observed in the Nyquist plots. Its diameter, corresponding to the interfacial resistance, is small for 0.1M Pip_13_DFOB LP30 solution rather than pure LP30. In addition, when LP30 is used, the shape of the semicircle is elliptic suggesting that the interphase between KS6 and LP30 is more resistive and less capacitive. For the symmetric cells with Li, similar semicircles are observed in the Nyquist plots. As Li metal has a strong reducing property, when electrolytes are not chemically stable, continuous increase in interfacial resistances occurs [23]. However, in the present case, the interfacial resistances are converged to 100 Ω, suggesting that both electrolytes are chemically stable enough for combining with the Li metal anode. Regarding the LNMO|LNMO symmetric cells, the semicircle shapes are not observed, and only diffusion resistances are visible. With the presence of the IL in LP30, resistance and capacitance of diffusion in the electrode become smaller. Overall, the addition of the IL is confirmed to be effective to form electrode-electrolyte interface with low resistances compared to pure LP30.

### 3.4. Galvanostatic Performance

**Figure 6:**
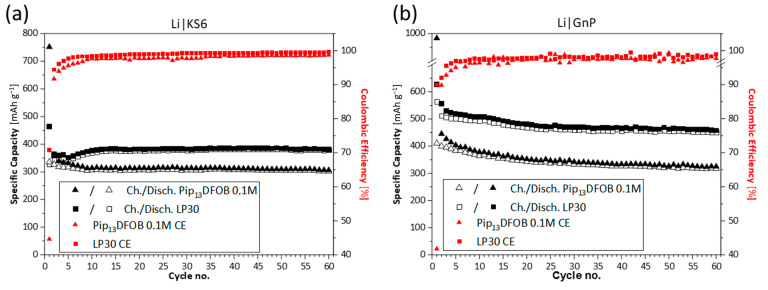
Galvanostatic cycling trends at 0.1C in Li|C, where C = KS6 (**a**) and C = GnP (**b**).

In order to analyze the effect of the IL in LP30 for battery application, the capacity trends over cycling were evaluated. The cycling performances of Li|C cells are reported in Figure 6. Compared to the cells with pure LP30, the capacity values obtained for the IL-added mixture are about 17% lower for the KS6 (305 mAh g^−1^ vs. 368 mAh g^−1^ after 60 cycles) and about 25% lower for the GnP (363 mAh g^−1^ vs. 486 mAh g^−1^ after 60 cycles). A favorable effect in terms of the capacity values is not observed by adding the IL. One reason can be the reduced relative concentration of Li^+^ in the solution and lowered ionic conductivity with the presence of the IL (see Figure 4) although their differences are small.

**Figure 7:**
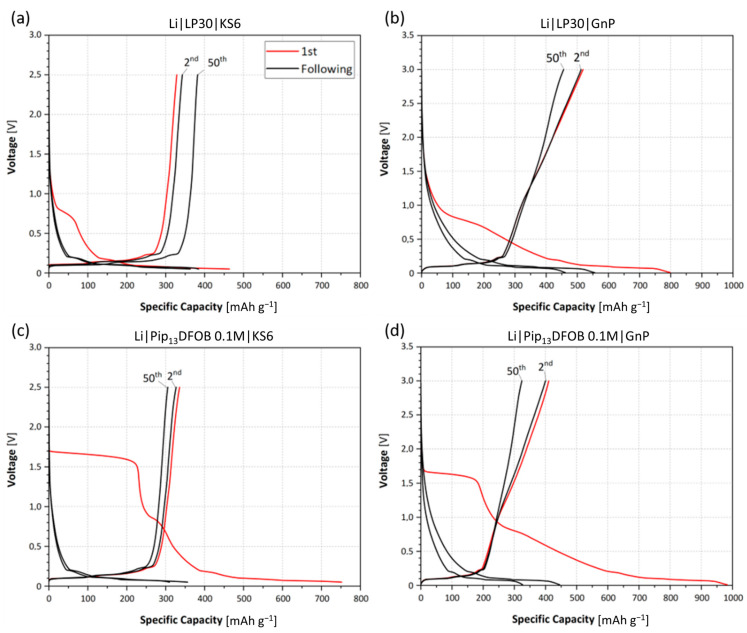
Voltage profiles of the 1st, 2nd, and 50th cycle of Li|KS6 cell without (**a**) and with (**c**) the addition of the IL and of Li|GnP cell without (**b**) and with (**d**) the addition of the IL.

Figure 7 summarizes voltage profiles of the aforementioned cells. In the first cycles shown in red, the effect of the IL is clearly visible. It is well-known that LP30 forms the SEI around 0.8–0.7 V vs. Li^+^/Li [24], and this reaction is visible as a shoulder starting from 0.8 V vs. Li^+^/Li during the first discharge. When the IL is present, the potential of the shoulder is raised to 1.7 V vs. Li^+^/Li. In the cyclic voltammetry of a similar electrolyte system, specifically carbonate-based electrolytes containing [DFOB]-based ILs, a typical decomposition reaction is observed at 1.4 V vs. Li^+^/Li [14]. Thus, the plateau at 1.7 V vs. Li^+^/Li is considered to be due to the reduction reaction and formation of SEI layer. Compared to pure LP30, the 1st Coulombic efficiency is low with the presence of the IL because of the amplified SEI formation as indicated by the wider potential plateau at 1.7 V vs. Li^+^/Li. The irreversible capacity related to the SEI formation appears only in the first cycle and almost disappears in the following cycles. Considering the lower capacity value of the cell with the IL, the SEI formed with the presence of [DFOB] anions is expected to be more resistive. Therefore, the addition of Pip_13_DFOB is less favorable for carbonaceous electrodes, which is probably related to the reduced ionic conductivity and the formation of resistive SEI.

**Figure 8:**
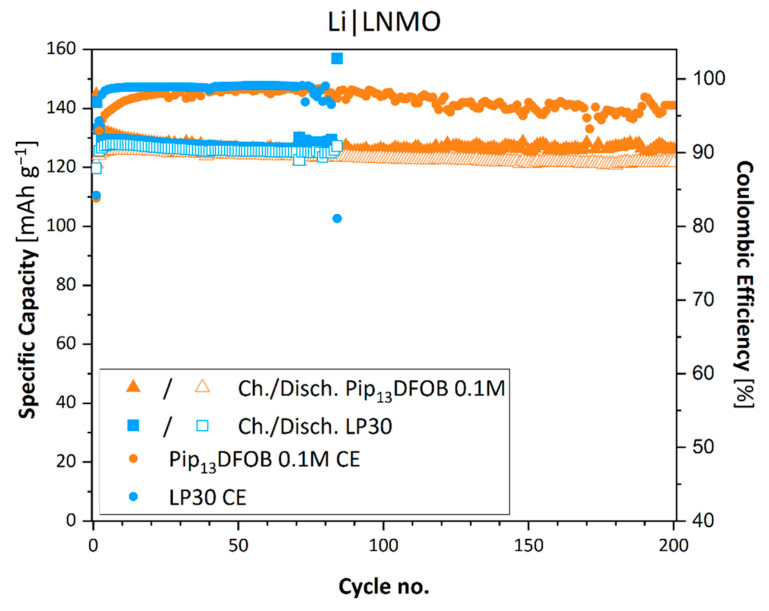
Galvanostatic cycling performances of Li|LNMO at 1C.

When the IL-added LP30 is used in a Li|LNMO configuration, a longer cycle life is achieved. Figure 8 shows the specific capacity of the cell in the range, where the Coulombic efficiency is maintained above 80%. Cells with both electrolytes show the specific capacity above 125 mAh g^−1^, reaching the nominal capacity reported by the supplier (130 mAh g^−1^). The voltage profiles at the 1st, 2nd, and 50th cycles are shown in the Supplementary Materials. Regardless of the presence of ILs, the cells exhibit a voltage plateau at around 4.7 V vs. Li^+^/Li. When Pip_13_DFOB is absent in LP30, the cell shows huge irreversible charge capacities after 80 cycles and its Coulombic efficiency falls below the definition. Since many spikes were observed in the voltage profile during charge (not reported), it is considered that the micro-scale Li dendrite formation occurs. In contrast to the fact that the cells with carbonaceous electrodes are cycled at 0.1C, the Li|LNMO cells are cycled at 1C; therefore, the cells have been cycled in conditions that accelerate the formation of lithium dendrites. When the same LNMO electrode was tested in our previous work, the spikes in the charge curve were not observed [14]. In this case, the cells have been prepared by using three pieces of Whatman separators, and, thus, penetrating dendrite formation was most likely limited by them. However, for higher energy density batteries, the number of separators should be reduced, and in the present case, only one piece of separator was used. This probably is not sufficient to avoid the formation of micro-scale short circuit. When the IL is present, the cell retains the Coulombic efficiency above 93% throughout 200 cycles. In addition, a good capacity retention is achieved by using the IL, with a value of 121.8 mAh g^−1^ at the 200th cycle. It is known that oxalate borate-based anions such as [BOB] and [DFOB] are able to form CEI that is rich in stable inorganic boron, fluorine, and carbonate compounds [14,25]. Taking this fact into consideration, the enhanced performance should be due to the formation of CEI on the LNMO surface. In addition, there is a possibility that the presence of [DFOB] somehow modifies Li metal surface and allows more stable lithium stripping–deposition phenomena with a lower risk of Li dendrite formation, when the cells are cycled at high C-rates. Therefore, it can be concluded that the addition of [DFOB]-based IL is not favorable for the cell working at lower potential, while it is noticeably crucial in case of high-voltage cells cycled with a higher speed.

## 4. Conclusions

In the present work, *N*-methyl-*N*-propylpiperidinium difluoro(oxalato)borate (Pip_13_DFOB) was synthetized. Fundamental physical–chemical characterizations, as well as electrochemical investigations, were carried out to elucidate the effect of the IL as an electrolyte additive. TGA shows a good thermal stability for the IL (>230 °C), and also confirms the absence of impurities from the synthesis. DSC reveals an endothermic peak due to IL melting at about 17 °C, defining our material as the RTIL. The IL shows an ionic conductivity around 1.24 × 10^−3^ S cm^−1^ at room temperature, about one magnitude lower than the commercial LP30 solution. By mixing 0.1M Pip_13_DFOB as an additive in a LP30 electrolyte, the ionic conductivity in the order of 10^−2^ S cm^−1^ is achieved. This mixture was tested in lithium metal cells versus different carbonaceous electrodes, specifically a KS6–graphite and GnP–graphene, and versus the LNMO high-voltage cathode. Impedance spectroscopy on symmetric cells suggests that the addition of the IL is effective in forming electrode–electrolyte interfaces with low resistance compared to pure LP30. Finally, despite the IL-added mixture performances in Li|C configuration being slightly lower than those of commercial LP30 electrolyte, remarkable improvement by the IL addition is observed in the Li|LNMO cells. Specifically, by adding the IL to LP30, the life of the cell is extended from 80 to 200 cycles, retaining good capacity (above 120 mAh g^−1^) and Coulombic efficiency (above 93%). It can be concluded that [DFOB]-based IL has not only a good thermal stability but also an ability to improve the cycle life of high-voltage batteries. This suggests that Pip_13_DFOB is a potential additive for the advanced LIBs, especially those characterized by higher working potential and higher cycling speed.

## Data Availability

Not applicable.

## Supplementary Materials

The following supporting information can be downloaded at: https://www.mdpi.com/article/10.3390/ma16041411/s1, Figure S1: NMR of Pip_13_DFOB; Tables S1–S3: Fitting results of Nyquist plots; Figure S2: Voltage profiles of Li|LNMO cell.
