# Supplementary material for: Effects of Difluoro(oxalato)borate-Based Ionic Liquid as Electrolyte Additive for Li-Ion Batteries
Source: Materials (Basel). 2023 Feb 8;16(4):1411. doi: 10.3390/ma16041411 (PMC9967885; doi:10.3390/ma16041411)
Supplement: Supplementary file 1 [file materials-16-01411-s001.zip › materials-2203795-supplementary.pdf]

Supplementary Materials

Effects of Difluoro(oxalato)borate-Based Ionic Liquid as Electrolyte Additive for Li-Ion Batteries

Graziano Di Donato, Giovanna Maresca, Matteo Palluzzi, Akiko Tsurumaki * and Maria Assunta Navarra *

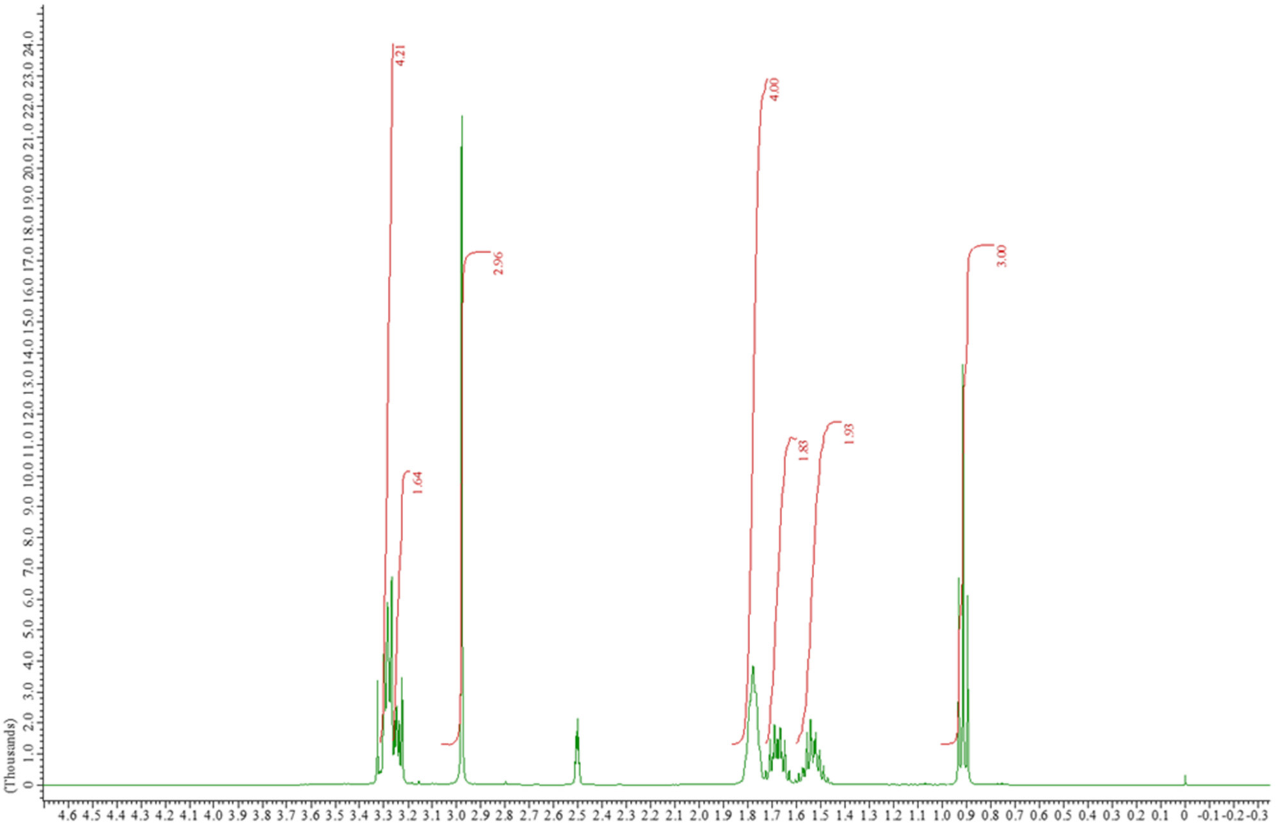

Figure S1. ^{1}H NMR of the synthetized Pip_{13}DFOB.

Table S1. Bulk resistance (R_b) and interfacial resistance (R_{int}) values of KS6|KS6 symmetric cells. The spectra shown in Figure 6 (a,b) were fitted by using an equivalent circuit of $R_b + Q_{int}/R_{int} + M$, in which R, Q, and M are resistance, constant phase element, and restricted linear diffusion element, respectively.

**Hour**	**LP30**		**LP30 + 0.1M Pip$_{13}$DFOB**	
	**R_b / Ω**	**R_{int} / Ω**	**R_b / Ω**	**R_{int} / Ω**
0	2.89	5.52	2.64	1.65
24	2.66	5.90	2.79	2.03
48	2.71	6.01	2.82	2.08
72	2.86	6.13	2.90	2.16
96	2.95	5.65	2.79	2.11
120	2.95	5.80	2.89	2.19
144	2.78	5.54	2.98	2.29
168	2.82	5.54	3.04	2.37

Table S2. R_b and R_{int} values of Li|Li symmetric cells. The spectra shown in Figure 6 (c,d) were fitted by using an equivalent circuit of $R_b + Q_{int}/R_{int}$.

Hour	LP30		LP30 + 0.1M Pip_{13}DFOB	
	R_b / Ω	R_{int} / Ω	R_b / Ω	R_{int} / Ω
0	5.04	89.97	6.28	43.11
24	5.11	71.62	6.95	64.42
48	5.14	74.87	7.22	64.18
72	5.14	90.04	7.29	78.73
96	5.03	101.40	7.18	101.00
120	5.90	66.22	7.16	68.33
144	5.00	63.11	6.92	84.18
168	4.62	83.29	6.98	78.34

Table S3. R_b values of LNMO|LNMO symmetric cells. The spectra shown in Figure 6 (e,f) were fitted by using an equivalent circuit of $R_b + Q_{dff}/W_{dff}$, in which in which W is Warburg element for diffusion.

Hour	LP30	LP30 + 0.1M Pip_{13}DFOB
	R_b / Ω	R_b / Ω
0	17.03	4.07
24	15.54	6.76
48	15.15	7.48
72	14.15	7.60
96	13.34	7.36
120	12.74	7.33
144	12.39	7.37
168	12.22	7.45

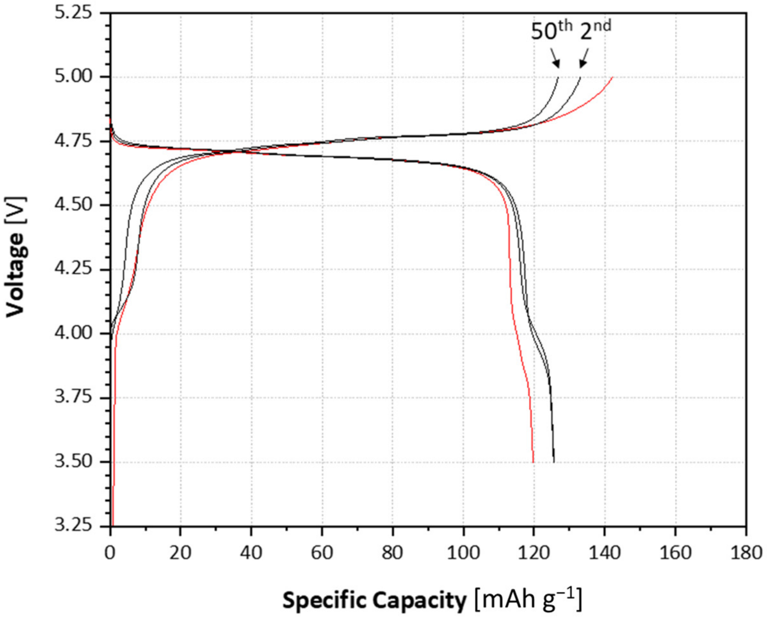

(a)

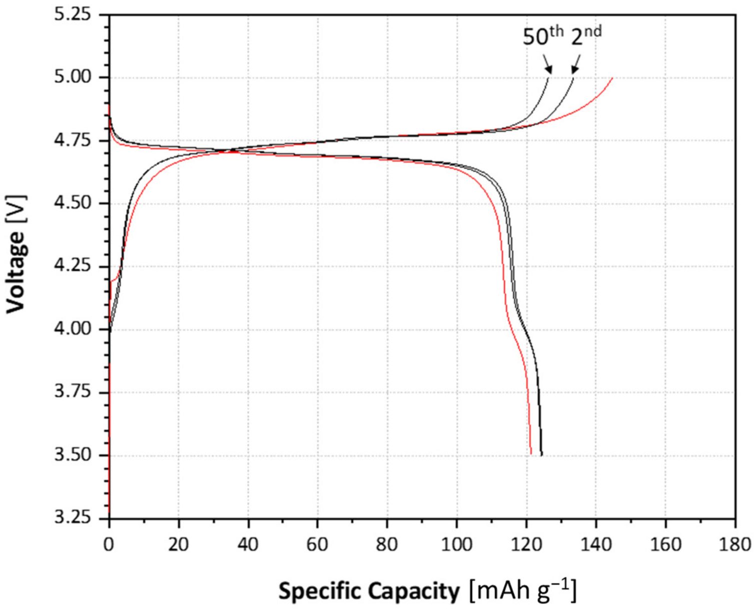

(b)

Figure S2. Voltage profiles of the 1st (red), 2nd and 50th (black) cycle of Li|LNMO cells without (a) and with (b) the addition of the IL.
